# TFIP11, CCNL1 and EWSR1 Protein-protein Interactions, and Their Nuclear Localization

**DOI:** 10.3390/ijms9081504

**Published:** 2008-08-25

**Authors:** Sissada Tannukit, Xin Wen, HongJun Wang, Michael L. Paine

**Affiliations:** University of Southern California, School of Dentistry, Center for Craniofacial Molecular Biology, 2250 Alcazar Street, CSA room 103, Los Angeles, California 90033-1004, USA. E-mail: paine@usc.edu

**Keywords:** Cyclin L1, Ewing’s sarcoma protein, nuclear speckles, pre-mRNA splicing, spliceosomal component 35, spliceosome, tuftelin-interacting protein 11

## Abstract

Previous studies using the yeast two-hybrid assay (Y2H) have identified cyclin L1 (CCNL1) and Ewing sarcoma breakpoint region 1 protein (EWSR1) as being interacting partners of tuftelin-interacting protein 11 (TFIP11). All three proteins are functionally related to the spliceosome and involved in pre-mRNA splicing activities. The spliceosome is a dynamic ribonucleoprotein complex responsible for pre-mRNA splicing of intronic regions, and is composed of five small nuclear RNAs (snRNAs) and μ140 proteins. TFIP11 appears to play a role in spliceosome disassembly allowing for the release of the bound lariat-intron. The roles of CCNL1 and EWSR1 in the spliceosome are poorly understood. Using fluorescently-tagged proteins and confocal microscopy we show that TFIP11, CCNL1 and EWSR1 frequently co-localize to speckled nuclear domains. These data would suggest that all three proteins participate in a common cellular activity related to RNA splicing events.

## 1. Introduction

Pre-mRNA splicing is an essential step of gene expression in many eukaryotic genes. The splicing activity is carried out by spliceosome, a multicomponent ribonucleoprotein complex containing five uridine-rich small nuclear RNAs (snRNAs; U1, U2, U4, U5, and U6) and a large number of associated proteins. In a recent comprehensive proteomic study using affinity selection and mass spectrometry, μ140 proteins were identified as components of the spliceosome [1]. Tuftelin-interacting protein 11 (TFIP11) has been identified as a spliceosome component in a number of proteomic studies [1–4]. TFIP11 is localized in a novel subnuclear compartment, termed the TFIP body, which is in close proximity to SC35 domains [5]. SC35 domains, also known as splicing factor compartments or nuclear speckles, are enriched in splicing factors essential for pre-mRNA splicing. The yeast homologue of TFIP11 is Ntr1/Spp382 (encoded by *YLR424W* gene in *S. Cerevisiae*) and shares about 30% amino acid similarity with mammalian TFIP11 proteins. The N-terminal region of TFIP11 contains a G-patch, which is a highly conserved domain of many RNA-processing proteins. In yeast, Ntr1 has been shown to interact directly with Prp43, an ATP-dependent RNA helicase [6–8]. This interaction results in the recruiting of Prp43 to the spliceosome, and is a required step for the release of the lariat-intron and spliceosome disassembly [9]. A number of TFIP11-interacting proteins involved in RNA processing have also been identified in a yeast two-hybrid (Y2H) screening of a mouse embryonic cDNA expression library [10]. These included cyclin L1 (CCNL1), DEAD box polypeptide 47 (Ddx47), Ewing sarcoma breakpoint region 1 (EWSR1), and polyA-binding protein cytoplasmic 1 (Pabpc1) [10]. Of these TFIP-interacting proteins, a number of molecular-based studies have identified CCNL1 and EWSR1 as being either involved, or potentially involved in RNA processing [11–14].

CCNL1 belongs to the cyclin family that contains C-terminal arginine and serine-rich (RS) domain, and a characteristic cyclin box [14]. The RS domain is a distinctive feature of the serine/arginine family of splicing factors required at various steps of spliceosome assembly and regulated splicing [15]. CCNL1 localizes to the nuclear speckles, subnuclear domains believed to be storage/assembly/modification sites for pre-mRNA splicing factors. Recently, CCNL1 was characterized as a splicing factor involved in alternative splicing regulation of the E1A minigene [11, 12].

EWSR1 is associated with various cancers that involve chromosomal translocation, either with genes of the erythroblastosis virus-transformed sequence (ETS) family, or with other known transcription factors [16]. EWSR1 has been shown to interact with integral components of the transcriptional complex [17], the splicing factor 1 (SF1) [18] and the U1 snRNP-specific protein C (U1C) [19]. These data imply that EWSR1 has dual activities that span gene transcription and RNA splicing [13].

In this study, we reported the intracellular colocalization of endogenous TFIP11 with fluorescently tagged CCNL1 and EWSR1 fusion proteins. Data presented here suggest that TFIP11, CCNL1 and EWSR1 each participate in a common cellular activity related to the events of RNA processing.

## 2. Experimental Section

### 2.1. Northern blot analysis

Northern Blot analyses were done using the FirstChoice Northern Human Blot 2 for multiple organs (Catalogue # AM3141, Ambion, Inc., Applied Biosystems, Austin, TX). Full-length cDNAs for human TFIP11, CCNL1 and EWSR1 were purchased from Origene (Rockville, MD; catalogue #s SC112654/NM_012143, TC113160/NM_020307 and TC116868/NM_005243 respectively). The 3’ terminal regions of TFIP11, CCNL1 and EWSR1 cDNAs were released with Eco RV and Xba I, Xba I and Apa I, Xba I (which cut 2 times) respectively to give partial cDNAs of ∼ 420 bp, ∼ 380 bp and ∼ 500 bp which were gel purified and subsequently used as the template DNA for the radiolabeled probe. Note that for all three partial cDNAs, the Xba I site at the 3’ terminus was part to the multicloning site of the host vector. The complete, 1.8 kb human β-actin (ACTB) cDNA was purchased from Clontech (catalogue # 636828) and used as a control. Double-stranded cDNAs were labeled with [α-^32^P]-dCTP using the Prime-It II Random Prime Labeling Kit (Stratagene, catalogue # 300385). Northern blot analysis was performed using standard methodologies [20].

### 2.2. Plasmid constructs

The TFIP11 cDNA, covering the entire open reading frame (ORF), was released from the construct TFIP11-N1 [5], and subcloned into the vector pCMV-3Tag-8 (Stratagene, La Jolla, CA) using appropriate restriction enzyme sites. The resulting construct is referred to as TFIP-FLAG.

Full-length human CCNL1 cDNA was purchased from Origene (Rockville, MD). The entire ORF of CCNL1, covering 526 amino acids and minus its stop codon, was amplified by PCR using the forward primer 5’-GATATCAAGACTATGGCGTCCGGGCCTC; and the reverse primer 3’-GGCGCCTGTGCCTGCCATGTCCTG. The CCNL1 start codon is underlined. The PCR product was directionally subcloned into the vector pcDNA3.1/CT-GFP-TOPO (Invitrogen Corporation, Carlsbad, CA) and the resulting plasmid is called CCNL1-N1.

For EWSR1, a full-length human cDNA was generated by RT-PCR using total RNA from a human lung tumor (Clontech catalogue # 636633). The primers used for PCR were forward 5’-TCTCGAGGAGAAAATGGCGTCCACGGATTACAG; and reverse 5’-TCCGCGGGTAGGGCCGATCTCTGCGCTCC which included an Xho I and a Sac II restriction site respectively (underlined). The purified PCR product was then directionally subcloned into the vector pDsRed-Express-N1 (Clontech) at the Xho I and Sac II multicloning site. The resulting plasmid is called EWSR1-Red-N1. The Myc-tagged EWSR1 vector was prepared by removing the EWSR1 cDNA from EWSR1-Red-N1 using Xho I and Sac II restriction enzymes, and subcloning this cDNA into pcDNA3.1/*myc*-His plasmid (Invitrogen Corporation) at the Xho I and Sac II multicloning site. This construct is called EWSR1-Myc.

All plasmid construct cDNA inserts were subcloned by standard methodologies [20], and sequenced in their entirety to verify that no nucleotide errors were introduced during their synthesis, and that the inserts were in the correct orientation and reading frame.

### 2.3. Cell culture and transient transfection

HEK293, HeLa, and LS8 cells [5] were maintained in Dulbecco’s modification of Eagle’s medium (DMEM) with high glucose (4.5 g/l) supplemented with 10% (v/v) fetal calf serum (FCS). For transient transfection assays, cells were grown on either four-well chamber slides (Lab-Tek) or 100-mm dishes. Lipofactamine 2000 (Invitrogen Corporation) was used as the transfection reagent according to the manufacturer’s instruction.

### 2.4. Immunofluorescence assay and confocal imaging

Cells were fixed with 4% paraformaldehyde for 5 minutes at room temperature (RT), permeabilized with 1% Triton X-100 for 10 minutes at RT, and washed with phosphate buffered saline (PBS). Cells were then incubated with primary antibodies for 1 hour at RT, washed with PBS three times, and incubated with secondary antibodies for 1 hour at RT. After incubation, cells were washed with PBS three times and mounted with VECTASHIELD medium (Vector Labs, Burlingame, CA). Confocal images were captured as previously described [5]. The primary antibodies were mouse anti-SC35 (Sigma-Aldrich, St. Louis, MO) and rabbit anti-TFIP11 raised against the peptide LQNEFNPNRQRHWQ (Zymed Laboratories Inc, South San Fransisco, CA). Secondary antibodies were Texas Red-conjugated goat anti-mouse, Alexa Fluor 488 goat anti-mouse and goat anti-rabbit and Alexa Fluor 568 goat anti-rabbit antibodies (Molecular Probes, Invitrogen Corporation).

### 2.5. Immunoprecipitation assay and western blot analysis

Transfected cells were lysed with RIPA buffer (10 mM Tris-HCl pH 7.6, 150 mM NaCl, 1% NP-40, 1% sodium deoxycholate, 0.1% SDS and a protease inhibitor cocktail). The cells were collected and sheared by passing through a 22-guage needle repeatedly. The cell suspension was collected by centrifugation at 14,000 rpm for 10 minutes. After centrifugation, the protein concentration of the supernatant was measured using a Bio-Rad protein assay kit (Bio-Rad, Hercules, CA). The cell lysate was pre-cleared by incubation with protein G agarose beads (GE Healthcare, Piscataway, NJ) for 1 hour at 4°C which was included to reduce the non-specific background. After pre-clearing, the cell lysate was incubated for 2 hours at 4°C with an anti-FLAG antibody conjugated to agarose beads (Sigma-Aldrich) using constant motion. Immunoprecipitates were then collected by centrifugation at 2,500 rpm for 5 minutes and washed three times with high-salt wash buffer (1M NaCl). The final pellet was resuspended in 2x SDS loading buffer, boiled for 4 minutes, and stored at −20°C. For Western blot analysis, immunoprecipitates and cell lysates were resolved by SDS-PAGE and transferred to Immobilon-P membrane (Millipore, Billerica, MA). The membranes were incubated with anti-FLAG or anti-Myc antibodies. The protein-antibody complexes were visualized by enhanced chemiluminescence (Amersham Biosciences, GE Healthcare, Piscataway, NJ).

## 3. Results

### 3.1. TFIP11, CCNL1 and EWSR1 Northern blot analysis

**Figure 1:**
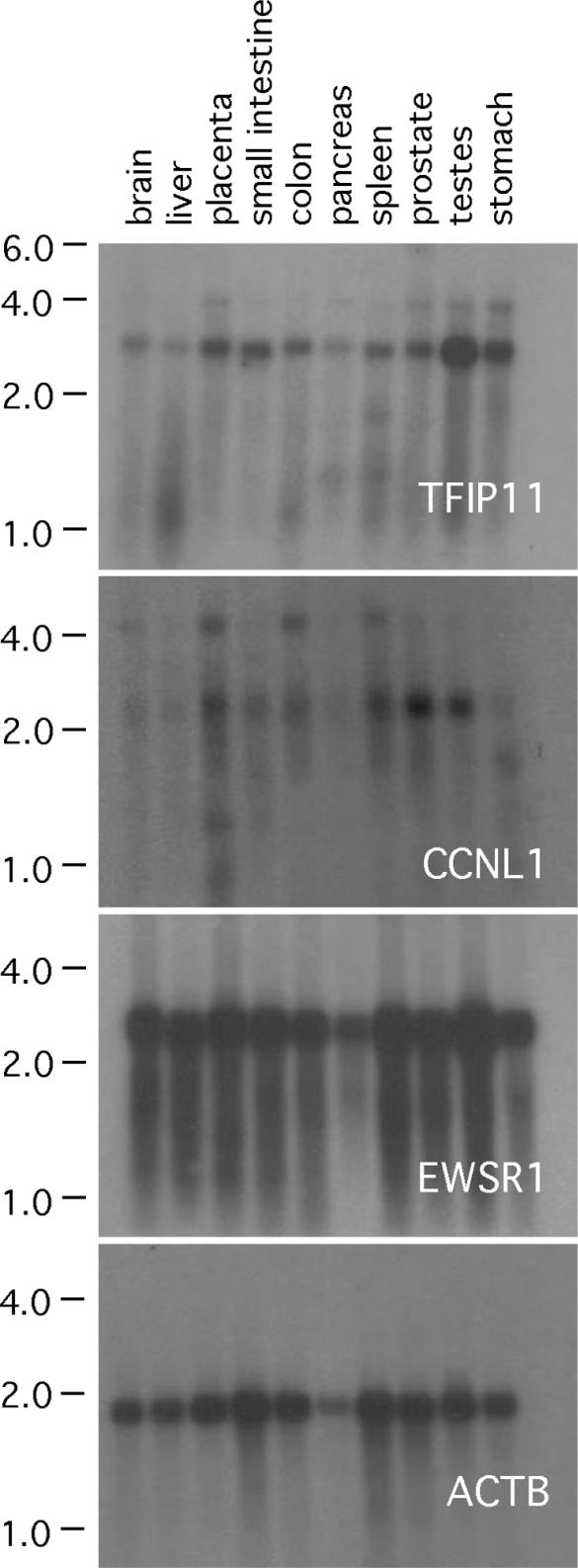
Northern blot analysis. Northern blot analysis of TFIP11, CCNL1 and EWSR1 in multiple organ tissues. Size markers in kb are shown on the left.

Northern blot analysis was used to characterize TFIP11, CCNL1 and EWSR1 mRNA expression profiles in multiple tissues. In all tissues tested, a signal was observed corresponding, in size, to the predominantly expressed mRNA transcript (Figure 1). For TFIP11, the predominant band corresponds to the predicted size (2.8 kb) of TFIP11 mRNA. In some tissues a weaker band at ∼ 4 kb is also apparent, and likely results from weak interaction with a non-related gene transcript. For CCNL1, 2 transcripts were noted at ∼ 2.3 kb and ∼ 4.5 kb. As discussed previously, the 2.3 kb band corresponds to the CCNL1 (also known as cyclin L α isoform) of 526 amino-acids, while the 4.5 kb band corresponds to an alternatively spliced CCNL1 transcript called cyclin L γ [14, 21]. For EWSR1, the transcript was observed at ∼ 2.6 kb. β-actin was used as a control.

### 3.2. Subcellular localization of GFP-tagged CCNL1 and RFP-tagged EWSR1

**Figure 2:**
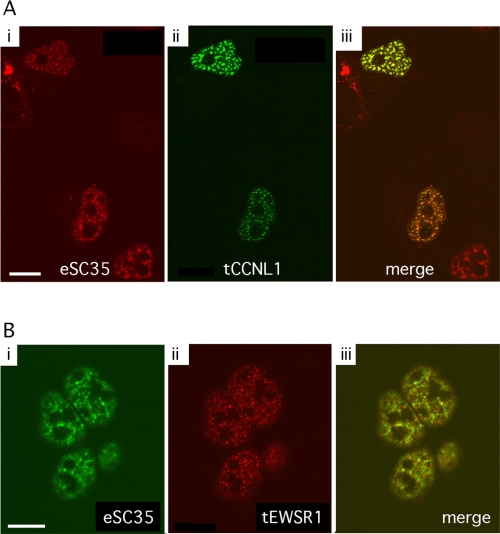
Subcellular localization of CCNL1 and EWSR1. LS8 cells transfected with CCNL1-N1 (panel A) or EWSR1-Red-N1 (panel B) and were fixed and labeled with anti-SC35 antibody. Endogenous SC35 (eSC35) localization (using Texas Red conjugated goat anti-mouse antibody; panel Ai) was compared to transfected CCNL1 (tCCNL1) localization (panel Aii) in the merged image (panel Aiii). Endogenous SC35 localization (using Alexa Fluor 488 goat anti-mouse antibody; panel Bi) was compared to transfected EWSR1 (tEWSR1) localization (panel Bii) in the merged image (panel Biii). Scale bars, 10 μm.

The splicing factor SC35 is a well-characterized molecular marker for the nuclear speckles. Nuclear speckles are subnuclear compartments for most, but not all, splicing factors. To visualize the subcellular localization of CCNL1 and EWSR1, compared to SC35 nuclear speckles, fusion constructs were generated with CCNL1 fused to green fluorescent protein (CCNL1-N1) and EWSR1 fused to red fluorescent protein (EWSR1-Red-N1). Each construct was transfected into LS8 cells individually. Consistent with previous studies [22], GFP-tagged CCNL1 showed discrete, punctate nuclear localization that corresponds to SC35 nuclear speckles (Figure 2A). Like CCNL1, EWSR1 also displayed a speckled pattern of expression within the nucleus (Figure 2B, ii). There was some, but not a significant amount, of colocalization between SC35 and EWSR1 (Figure 2B, iii). SC35, CCNL1 and EWSR1 all showed speckled nuclear localization, and in all cases expression is absent in the nucleoli. SC35 and CCNL1 showed a high degree of colocalization.

### 3.3. Colocalization of TFIP11 and CCNL1, and TFIP11 and EWSR1

**Figure 3:**
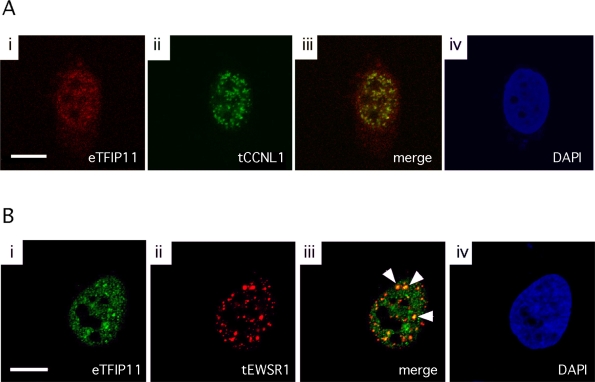
Colocalization of TFIP11 and CCNL1, and TFIP11 and EWSR1. HeLa cells transfected with CCNL1-N1 (panel A) or EWSR1-Red-N1 (panel B) and were fixed and labeled with anti-TFIP11 antibody. Endogenous TFIP11 (eTFIP11) localization (using Alexa Fluor 568 goat anti-rabbit antibody; panel Ai) was compared to transfected CCNL1 (tCCNL1) localization (panel Aii) in the merged image (panel Aiii). Endogenous TFIP11 localization (using Alexa Fluor 488 goat anti-rabbit antibody; panel Bi) was compared to transfected EWSR1 (tEWSR1) localization (panel Bii) in the merged image (panel Biii). Cell nuclei are stained blue by DAPI (panels Aiv and Biv). Scale bars, 10 μm.

CCNL1-N1 and EWSR1-Red-N1 were used to study the subcellular localization within the cell nucleus, and related to the spatial nuclear expression pattern of endogenous TFIP11. HeLa cells transfected with either CCNL1-N1 or EWSR1-Red-N1 were immuno-labeled with the anti-TFIP11 antibody (Figure 3A, i and Figure 3B, i). TFIP11 was shown to localize to distinct nuclear speckled domains, and was excluded from the nucleoli as previously demonstrated using a fluorescently-tagged TFIP hybrid protein [5]. CCNL1-N1 and EWSR1-Red-N1 also showed a nuclear speckled expression profile that did not extend into the nucleoli (Figure 3A, ii and Figure 3B, ii respectively). The nuclear distribution of CCNL1 frequently showed some degree of colocalization with TFIP11 (Figure 3A, iii), while EWSR1 more frequently was observed to colocalized with TFIP11 in the nucleus (indicated by arrowheads, Figure 3B, iii). The cell nuclei are identified using DAPI (4’-6-Diamidino-2-phenylindole) staining (Figure 3A, iv and Figure 3B, iv). These data suggest an apparent stable physical relationship between TFIP11 and CCNL1, while perhaps a fleeting or transient relationship exists between TFIP11 and EWSR1.

### 3.4. EWSR1 interacts with TFIP11 in HEK293 cells

**Figure 4:**
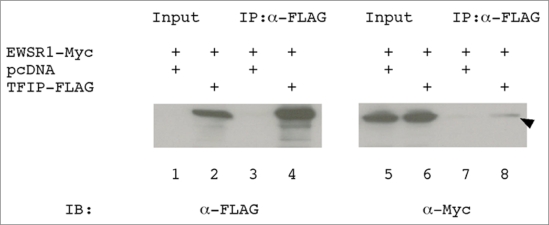
EWSR1 interacts with TFIP11. HEK293 cells were transfected with plasmid expressing EWSR1-Myc (lanes 1, 3, 5 and 7), or EWSR1-Myc and TFIP-FLAG (lanes 2, 4, 6 and 8). The input panels (lanes 1, 2, 5 and 6) show protein in the cell lysate prior to immunoprecipitation. Cell lysates were immunoprecipitated using anti-FLAG monoclonal antibody (lanes 3, 4, 7 and 8), and then immunoblotted with either the anti-FLAG (lanes 1–4) or anti-Myc antibody (lanes 5–8). The plasmid pcDNA was cotransfected as a blank control to ensure equal concentrations of plasmid DNA being used for each transfection. Immunoprecipitate (IP), Immunoblot (IB).

We previously reported the interaction between TFIP11 and CCNL1, as well as, TFIP11 and EWSR1 using Y2H assay [10]. To verify the biological relevance of data from yeast two-hybrid system, we performed coimmunoprecipitation assay in HEK293 cells. Cells were transfected with EWSR1-Myc or cotransfected with EWSR1-Myc and TFIP-FLAG. When immunoprecipitated with anti-FLAG antibody, EWSR1 was coprecipitated only when TFIP11 is present in the cell lysate (Figure 4, lanes 7 and 8). This *in vitro,* coimmunoprecipitation data demonstrated the interaction between TFIP11 and EWSR1 (arrowhead; Figure 4, lane 8). We were unable to detect the interaction between TFIP11 and CCNL1 by coimmunoprecipitation. Using coimmunoprecipitation we have been able to confirm the TFIP11-EWRS1 interaction previously shown by the Y2H assay, but have been unable to demonstrate an interaction between TFIP11 and CCNL1. The failure to show a TFIP11-CCNL1 interaction by coimmunoprecipitation may relate to the relative strengths of the protein-protein associations, or may relate to the relative sensitivities of the *in vitro* based assay, as compared to the *in vivo* derived, Y2H data.

## 4. Discussion

In this study we have focused our attention on the spatial relationship of three proteins related to the pre-mRNA splicing: those being TFIP11, CCNL1, and EWSR1. Published data have identified each of these proteins as having a nuclear localization, and each has functional domains that identify them as being associated with RNA processing. Here we report subcellular colocalization between TFIP11 and its interaction partners, CCNL1 and EWSR1, which were previously identified by a Y2H library screening [10]. Using coimmunoprecipitation, we have confirmed the Y2H data identifying TFIP11 and EWSR1 interaction but have been unable to coimmunoprecipitate TFIP11 and CCNL1.

The current paradigm of spliceosome assembly is that spliceosome assembles by sequential interaction of U1, U2, and U4/U6.U5 tri-snRNP with the pre-mRNA, creating unique short-lived intermediates of the spliceosome, designated E, A, B, and C complexes [2, 23]. Recent proteomic analyses have identified TFIP11 and EWSR1 as components of spliceosome. TFIP11 was identified within complexes B* [3] and C [23], EWSR1 within complex C [2, 23], SC35 within complex A [24].

We have previously described TFIP11 as localizing to subnuclear structures in close proximity to SC35 domains [5]. SC35 belongs to the SR protein family of splicing factors having a modular structure that contains one or two N-terminal RNA recognition motifs and a C-terminal RS domain. The SR proteins primarily localize to the nuclear speckles, subnuclear domains largely corresponding to the interchromatin granule clusters, which contain little or no DNA at the level of electron microscopy [25]. Photobleaching experiments revealed that CCNL1 is an immobile component of splicing factor compartments [22]. Thus, our data demonstrating TFIP11 and CCNL1 colocalization suggests that TFIP11 resides in close proximity to nuclear speckles and/or may act as a subnuclear storage compartment for splicing components as previously suggested [5].

The intracellular distribution of EWSR1 showed punctate subnuclear structures resembling the nuclear speckles. It was previously reported that EWSR1 colocalizes with the survival motor neuron protein (SMN), which is a component of Cajal bodies [26]. Cajal bodies are dynamic nuclear structures involved in snRNP biogenesis [26]. Despite the fact that EWSR1 interacts with the splicing factors SF1 and U1C, which both function at an early stage of spliceosome assembly, native EWSR1 has no apparent impact on the alternative splicing regulation of the E1A minigene [27]. TFIP11 modulates splice site selection in the E1A pre-mRNA splicing assay, suggesting that TFIP11 is involved in alternative splicing regulation [5]. It is therefore unlikely that the interaction between TFIP11 and EWSR1 contributes to the regulation of alternative splicing.

Our results indicate the similar spatial expression pattern between TFIP11 and CCNL1, and a less-well defined spatial overlap between TFIP11 and EWSR1. These finding are complementary to previously published Y2H data showing protein-protein interactions between TFIP11 and CCNL1, and between TFIP11 and EWSR1. The functional relationship of these three proteins remains to be determined. While the functional spliceosome appears to consist of ∼140 proteins, future proteomic analyses (experimental and *in silico*) will be able to establish which of these splicing proteins are redundant, and which are absolutely essential. Identifying protein-protein interactions between the various splicing factors, and relating these to their spatiotemporal expression profiles *in vivo*, will lead to a better understanding of the molecular mechanism of RNA splicing.
